# The emotional labour of quality improvement work in end of life care: a qualitative study of Patient and Family Centred Care (PFCC) in England

**DOI:** 10.1186/s12913-019-4762-1

**Published:** 2019-12-02

**Authors:** Richard Boulton, Annette Boaz

**Affiliations:** Centre for Health and Social Care, St George’s, University of London and Kingston University, CHSCR, 6th Floor, Hunter wing, London, SW17 0QT UK

**Keywords:** Shadowing, PFCC, Workforce capacity building, Patient experience, Emotional labour, Dying, Bereavement, End of life care

## Abstract

**Background:**

There is a growing emphasis on understanding patient experience in order to inform efforts to support improvement. This paper reports findings from an implementation study of an evidence-based intervention called Patient and Family Centred Care (PFCC) designed to tap into patient experiences as a basis for improvement. In this study the PFCC intervention was spread to a new service area (end of life care) and delivered at scale in England. The findings presented here focus specifically on one key aspect of the intervention: staff shadowing of patients, and the experiences of staff carrying out shadowing for the purposes of service improvements.

**Methods:**

The study methods were ethnographic observations of key events, semi-structured interviews with members of participating teams and the programme implementation support team and managers, and a review of the documents used in the set up and running of the programme.

**Results:**

One of the key strengths of the PFCC approach is to encourage staff through shadowing to engage with patient experience of services. Many staff described the process of shadowing as a transformative experience that alerted them to immediate areas where their services could be improved. However, engaging with patient experience of end of life care services also had unintended consequences for some staff in the form of emotional labour. Furthermore, we observed difficulties encountered by staff that are not accounted for in the existing PFCC literature relating to how care service structures may unevenly distribute the amount of ‘emotional labour’ that staff members need to invest in implementing the programme.

**Conclusions:**

Connecting with patient experience is a crucial aspect of a number of quality improvement interventions that aim to help staff to engage with the lived experience of their services and reconnect their motivations for working in the health care system. However, there may be unintended consequences for health care service staff, particularly in sensitive areas of service delivery such as end of life care. The ‘emotional labour’ for staff of engaging in quality improvement work informed by patient experience should be considered in planning and supporting patient experience led quality improvement.

## Background

Transforming health care organisations through quality improvement work is gaining considerable attention in the UK NHS and in other countries (Eccles et al., 2009; Glasgow et al., 2012). A wide range of tools and techniques have emerged to support quality improvement activities (Greenhalgh et al., 2004; Nilsen, 2015). In particular, there is a growing emphasis on patient experience as a means to build capacity and support improvement (Bate and Robert, 2006; Callard and Rose, 2012; Rose, 2014). In 2015 the Point of Care Foundation^1^ received funding from the UK Health Foundation Spreading Improvement programme, and set up a programme titled: ‘Living well to the Very End’. In this programme the Point of Care Foundation used an evidence-based approach called Patient and Family Centred Care (PFCC) to support improvement. PFCC was originally developed by the Innovation Centre at the University of Pittsburgh Medical Centre [1]. It has subsequently been adapted and refined by the Point of Care Foundation team through its application in previous improvement programmes. The programme has six core elements^2^ designed to establish infrastructure for improvement, understand the current and ideal state of the service and work to deliver improvements. This article will focus on one of these core elements; patient shadowing. As detailed in the original PFCC literature from Pittsburgh by DiGioia and colleagues [1], shadowing is designed to be a transformative experience for health care service staff, where practitioners see the service from the perspective of patients (and their families) and in the process not only see the problems with their service but are also able to empathise from the perspective of the patient and possibly pre-empt their reactions to receiving care and build service capacity in response. The programme originators describe the importance of shadowing as follows:

“Perhaps more than any other segment of the experience improvement process, shadowing, and care flow mapping created a sense of empathy and urgency among caregivers by highlighting and clarifying the patient and family experience in a way that cannot be understood unless one “walks in their footsteps.” Brown (2009) writes in ‘Change by Design: How Design Thinking Transforms Organizations and Inspires Innovation’ that observation leads to empathy, which, in turn, leads to a sense of urgency and action; empathy “is how we translate observations into insights.” Traditional methods of asking people how to improve a service (e.g., focus groups and surveys) seem simple and logical, and they do have a role in PFCC; yet such methods usually lead to incremental improvements rather than insights that lead to innovation and transformation [2].”

The Point of Care Foundation identified end of life care as a priority topic that could benefit from PFCC improvements. In particular, it was felt that there was a degree of uncertainty about how best to deliver good quality end of life care, along with a new policy response from NHS England detailing the ambitions for palliative and end of life care going forwards (National [3, 4]).

The goals of the ‘Living Well to the Very End’ programme were to develop and apply PFCC by:

- Building capacity and capability in patient-centred quality improvement using PFCC methods across the English NHS
- Demonstrating the utility of PFCC methods for improving end of life care
- Developing an on-going mechanism for spread of expertise in PFCC methods nationally
- Contributing to the body of knowledge about scaling up health care innovations.

The programme was delivered in two phases through four workshop style learning events where service teams would receive training and guidance on the intervention. In the first phase the Point of Care Foundation team worked with local teams to implement PFCC in end of life care in eight acute and community settings in the South of England. In the second phase the team provided a lighter touch support with additional peer coaching to sixteen end of life care teams across a wider range of settings including care homes and hospices as well as acute settings across England. At the end of each phase of the programme participants came together for a celebration event where they shared their services improvements. In total the programme ran for 3 years from 2015 to 2018.

To evaluate the programme an independent research team comprising the two authors of this paper was commissioned. This paper presents findings from the independent evaluation of the programme. It focuses in particular on the impact of shadowing on the service staff carrying out the implementation work. In order to frame analysis of the data this paper draws upon wider literature from the social sciences to expand the existing literature on evaluating improvement interventions and implementation science initiatives. Literature from the sociology of dying and bereavement argues that death in contemporary society is often sequestered and hidden away behind the walls of the hospital or hospice [5, 6]. This sequestration often means that the struggles of caring for dying people and supporting people facing loss and grief before and after death are often over looked ([7]; Hospice Friendly Hospitals [8, 9]). As a result the challenges of end of life care may result in difficulties in delivering improvement programmes as intended as staff may need support and solicitude in ways other services may not. This opens up the question of the suitability of generalised improvement programs for use in sensitive service areas like end of life care. Emotional labour theory demonstrates how workforce strains can stem from incidences where emotions have been felt but not adequately expressed, resulting in emotional dissonance [10, 11]. Dissonance can occur when staff are being asked to change services or take on new responsibilities, particularly when undertaking such ventures tends to be unevenly distributed to certain individuals or groups of staff. We draw on emotional labour theory to consider if the use of improvement programmes may provoke sensitivities and insecurities in individuals and service structures [12, 13]. We discuss how the extent of emotional dissonance among staff may be even greater when improvement approaches are applied to sensitive service areas such as end of life care.

## Methods

The research set out in this paper originates from an evaluation of the PFCC programme, and uses data collected in the evaluation to explore the use of shadowing in end of life care services. The overall approach of the evaluation was ethnographic. Ethnography can contribute to process evaluation by providing rich accounts of activities, projects and programmes (Hammersley and Atkinson 2007, Vougioukalou et al. 2018). Within the ethnography, interviews and documentary analysis were added to incorporated multiple methods and varied lines of inquiry, to achieve ‘within research’ and ‘between method’ triangulation (Denzin 1989). The evaluation team was supported by an evaluation steering group, drawing on expertise in implementation and improvement, evaluation and end of life care. The team was also in regular contact with the implementation team based at the Point of Care Foundation (who were delivering and supporting the programme).

The study collected data on both phases of the intervention. The first phase of the programme involved observations at the learning events for staff, analysis of project documents, semi-structured interviews with staff and focus groups. The research activities focused more heavily on four of the eight sites, in order to provide more in depth case studies of the sites with the resources available. The four sites were selected to achieve variation based on three criteria: types of health care setting, geographical spread within the programme area (South of England) and rural/ urban setting. Based on these criteria the sites were paired and one site selected at random from each pair. Four final sites were selected which have been anonymised here for ethical reasons. One site was excluded as the applicants had identified particular ethical issues related to its patient population. These included the need to minimise burden on patients linked to research participation and potential risks for the lone researcher conducting interviews in drug and alcohol service settings. The fieldwork at the four case study sites involved a combination of site visits, face-to-face interviews and telephone interviews. The wider applicability of the findings from the four sites was explored through telephone interviews with staff in the remaining four sites.

The evaluation of the second phase (where PFCC was used in sixteen further organisations) involved further event observations (including more informal interviews with event participants), document analysis and semi structured interviews with staff at four of the sites. Semi structured interviews were also conducted with the coaches who were supporting the teams and with the implementation team. Across the two phases, 36 interviews were conducted, over 116 h of observation were carried out, three site visits to phase one sites were completed and documents were reviewed from the extensive archive of over 700 project documents shared by the implementation team (see Appendix 1 for full details of the methods used, Additional files 1 and 2). The data were transcribed and uploaded into NVivo and then analysed using grounded theory techniques of open and axial coding. Grounded theory uses inductive reasoning to allow repeated ideas and concepts in qualitative data to be woven together to create hypotheses and theories pertaining to the main concerns for participants (Foley 2015). The themes that emerged were ‘the role of shadowing’, ‘emotional responses to shadowing’, ‘anxieties to shadowing’, and ‘resistance to shadowing, which is further broken down to personal and managerial resistance. The initial analysis was presented and discussed with the evaluation steering group at a one day workshop.

## Results

### The role of shadowing

From the very first learning event the importance of shadowing was emphasised by the Point of Care Foundation to participating teams. The booklet distributed at the start of the first session introduced shadowing to the teams:

*What is patient shadowing?*

*As part of the pre-work phase of the programme, we are asking you to understand where care systems and processes currently prevent staff from providing excellent care. Key to this is developing an understanding of what patients’ experience as they move along the pathway and through the hospital.*

*One way to do this is through partnering staff or volunteers with patients, following them through the care pathway from the beginning to the end and reporting on their experiences. From a practical point of view, this may involve shadowing different parts of the pathway with different patients.*

*Patient shadowing enables real-time feedback to be obtained from patients, it raises staff awareness of the patient experience and it helps staff to understand what is important to patients and their families, not just what staff think is important.*

*(PFCC learning event materials: learning event 1)*

When programme team members at the Point of Care Foundation were asked in interviews which part of the PFCC was essential the team consistently emphasised the shadowing component as the most important to the successful implementation of the PFCC:

*“Interviewer: In terms of when you think about Patient and Family Centred Care, what is it that you think is critical, that does worry you if they don't do?*

*I mean, shadowing.*

*Interviewer: Anything else?*

*The shadowing, definitely, because I think that is really the crux. That's what changes people's minds. The shadowing's really key to making sure that they get it, and that actually… All the teams turned up at the celebration event, and they said, 'We thought we were going to be working on X, Y and Z, and then we went through this process, and by actually eventually trusting in the process, we ended up working on completely different things.' It's so powerful, because we know that what we've worked on are the real priorities. We know that we've used that time really well, we've made a really good difference than if we'd just gone ahead and done things anyway. I know there's been a little bit of resistance. Some of the teams, they come and ask you, 'Well we work in the service every day? We know what the problems are, we know what it's like, we don't need to.' I worry about converting that kind of attitude, and unless they actually do the shadowing then they won't get it. I think that, definitely.” (Programme Team member 2)*

Therefore, one theme that figured largely in the evaluation of the programme is the role of shadowing in fulfilling the aims set out by the programme and the ways in which teams have dealt with and responded to shadowing. The aim of the programme was to enable practitioners to process and interpret care situations with empathy towards patients and families as needed and as they arise. To achieve this, shadowing was adopted as the key ‘mechanism driving change’. Effective shadowing should have the role of helping staff to “walk in patients’ shoes” and become aware of things that could improve services based on their observations. The following quotation provides an illustration based on a staff experience of shadowing a patient in his side room:

*“On one side, there's a staff toilet and on the other side there's a patient’s toilet! For a patient who is very unwell like that, very poorly, we would leave the door open, but it meant it was very noisy and the supper trolley came. Somebody brought a hot meal which was inappropriate, and I looked around the room when the nurses had left, the last IV bag was still there and it was really - we should have taken that out. It was very informative and I realised we needed to buy some proper door stops and not be propping the door open, and things like that. I was there, I was able to help give him some symptom management because he wasn't very comfortable. That was helpful for his care. It was very interesting, and it was very interesting watching the communication as well.” (Hospital 2, Practice Development Nurse)*

Clearly evident here is the potential of shadowing as a means to increase staff awareness of the services they are delivering. In order for shadowing to take effect care practices must stand out to staff when they have not previously. Or to put it another way, staff must feel what DiGioia (2010 p.541) describes as the ‘urgency and empathy’ and the transformative effect of shadowing. The following quote reflects the experience of shadowing as described by a staff member:

*“I think that's really where the shadowing helped. That really sort of helped open people's eyes. I think taking a step back and being able to look in on something that you don't normally look in on - you are in the throes of it - I think really helped the team identify where they thought things could improve in terms of patient experience. Hopefully over time, because it's come from within - and I think that's one of the fundamental things over the two years is trying to find your champion from within the team, as opposed to feeling like you're doing something on top of somebody. It very much is sort of grassroots grown up.” (Hospital 2, End of Life Nurse)*

Rather than describing shadowing as being a sudden and abrupt ‘lightbulb moment’ staff seem to describe shadowing as a more gradual process of becoming aware of their service in a new way.

### Emotional responses to shadowing

The overwhelming emotional resonance that staff experience when undertaking shadowing of end of life care services is a consistent theme in the evaluation findings:

*“I just thought, oh gosh, it is such a stripped-back experience. The poor man in his gown and his incontinence pad and you just feel… I felt it was very impersonal. The table was there and there was his watch and he did have a mobile phone. His watch and his mobile phone were on the table, and you think, he's not able to use those anymore and these are the bits of him as a person.” (Hospital 2, training lead)*

This emotional response to shadowing is one that was seen throughout this research and was demonstrated and reported back throughout the learning events. Staff often talked at the events about close personal experiences of family members who had passed away and the care they received. In addition, the emotional power of shadowing was cited by staff at events as initiating many transformative effects. By the end of the programme a celebration event was held where all participating services demonstrated successful improvements made. Some examples included: changing parking arrangements for family members visiting relatives receiving end of life care; reorganising various administrative procedures; assessing the visibility of sensitive wording around the ward; introducing the use of symbols such as butterflies to communicate sensitive information; and rearranging the layout and upkeep of rooms, waiting areas or family rooms (examples drawn from PowerPoint presentations and posters put together by participating teams and presented during the celebration event).

### Anxieties to shadowing

The findings point to several elements of shadowing that staff found caused anxiety, chiefly the concern that partaking in shadowing consisted of putting colleagues under scrutiny:

*“So the feedback process after we'd done the shadowing, I think that people felt quite exposed about what might come and what might be seen and what might be shared. There was a lot of nervousness around the shadowing, but people embraced it.” (Hospital 2, End of Life Nurse)*

For those expressing this concern, staff members were not always optimistic about shadowing and may have had some fear as to what to expect from shadowing. This was also compounded if staff were being asked to do shadowing by more senior members of a team.

*“Shadowing is the thing that generates massive anxiety and I remember being asked several questions about that.*

*Interviewer: what kinds of questions were you asked?*

*‘What were they asking? Consent. What happens if you see something that you're not comfortable with or isn't good practice? I suppose they were the things that come immediately to mind.” (Hospital 1, Consultant)*

Here we see the anxiety that shadowing caused to many staff members, especially in the face of service hierarchies. For example, the staff expected to do the shadowing were often junior nurses and administrative staff. One important question raised by this finding is the extent to which team dynamics play a part in how something like shadowing is received by those working in services (an observation which is increasingly made in the quality improvement literature [14]).

### Resistance to shadowing

A further challenge for the implementation team was that several staff members in the sites felt that they could not complete shadowing at all. A range of issues may have contributed to non-completion of shadowing. We have grouped these into two categories. The first involves ‘personal resistance’ to shadowing including some of the constraints whereby services felt it impossible to do shadowing. The second involves ‘managerial resistance’ to shadowing, reflecting the attitudes of some teams whereby they felt unable to shadow.

#### Personal resistance

In terms of personal resistance to shadowing, some staff members felt that it would not be practical for them to go out and complete shadowing:

*“Well, very early on we were talking about end-of-life care for people who lived complex lives, so we were talking about people with substance misuse issues, that might be homeless, that might be in and out of street homelessness as well as the hostel system. We felt that there was a range of challenges to our project around our personal safety, if we were shadowing people in some of those environments if they were drinking and drugging. I think we spent a lot of time, didn't we, going I'm not sure how helpful that would be, sitting on the street with street drinkers, having a shadowing experience, because we weren't focused in a hospital environment with a clear direction…quite quickly we decided that shadowing some of those service users probably would be quite risky.” (Hospital 5, Focus group participant)*

This point alludes to the complexity of shadowing and staff reluctance to try it. The way it is presented here is that the service would find it impractical to carry out the shadowing exercise. In addition to safety aspects, there was a concern that some of the service users may not feel comfortable with being observed (although for those who tried shadowing this assumption was found to be largely unfounded). The programme team acknowledged the difficulties with this service user group, but felt that there would be opportunities (for example when service users came for consultations) for staff to try shadowing. At the learning events the teams heard from previous sites where shadowing had been undertaken and were given an opportunity to work through how they might use shadowing in their setting. At one such event, a participant from an earlier PFCC cohort who had not done shadowing reflected that, seeing how others had successfully conducted shadowing in end of life care, her team had perhaps not been sufficiently open to trying the approach, assuming it would be too difficult in end of life care. She reflected that, if she had another opportunity she would try harder to include shadowing in improvement work. Introducing shadowing for some services can involve a process of adaptation and there continued to be a tension between the ways in which shadowing was presented as achievable and essential to the approach, and the ways services might need to adapt shadowing in order to be culturally, socially or politically acceptable.

#### Managerial resistance

The second category links to this point by suggesting not only that staff may find shadowing impractical, but also that managerial staff might demonstrate deeper resistance to undertaking shadowing. For example, at one site the team described their decision not to shadow:

*‘We didn't do any shadowing, we were very clear that we don't think that for us that would be an appropriate thing to do at the time. We'd looked at it and we looked at it quite seriously, but for us, shadowing end-of-life patients, and for us a lot of our patients choose to come to die in our service because we have known them for a very, very long period of time. We're a regional service, tertiary referral service, some of our patients travel quite a long distance to come to us after their families and we didn't feel it would be appropriate to put this added thing on to the families.’ (Hospital 3, Matron)*

This quote again highlights the difficulties around shadowing, particularly in contexts where staff have a strong feeling that they know their patients well and already have a good understanding of what they need in terms of service improvement. The perception of shadowing as an ‘added thing’ or burden for patients was not supported at other sites where shadowing was undertaken with patients. The concern is that in some cases staff may be missing an opportunity to see their services ‘afresh’ through shadowing and to challenge their perceptions with regard to what was best for the patients and what might be the more critical improvement priorities.

What is developed further here however, is the concern amongst many participants about the impact that shadowing can have upon services. Not only is shadowing a concern for those planning it, but also those being observed and those called to do it. The introductory booklet given out to participating service teams at the beginning of the programme clearly states that in order to ensure that an observation doesn’t “feel threatening and judgemental” care must be taken when carrying out shadowing (‘Phase 2 Booklet’ PoC learning resource). The message can get lost once teams begin to plan the logistics or carry out the observation. Shadowing in this context takes on an extra dimension of highlighting ethically charged and sensitive areas of the services seeking to use it as a tool.

As a result, despite the emphasis on shadowing in the programme materials, training sessions and support visits and calls, in both the first phase and the second phase of the research several sites had not completed any shadowing activities and many of the sites that had completed shadowing had done so partially with the task of shadowing being done by more junior staff. For example, staff reported difficulties in persuading more senior colleagues to undertake shadowing.

## Discussion

It can be argued that the emotional impact of observing services from the perspective of the patient makes shadowing a powerful mechanism for service improvement [15] and a range of examples were shared at the celebration event. However, our findings suggest that asking staff to use PFCC style shadowing may also intensify the experience of providing end of life services and therefore constitute staff fulfilling a form of ‘emotional labour’. Through carrying out the PFCC in end of life care, staff may be being asked to face death in ways in which they may not feel comfortable. Improvements may ask staff to confront head on the emotional dissonance of providing care in the circumstances end of life care requires [16]. This may therefore result in the kinds of resistance and anxiety seen in the data [17–19]. The success of PFCC therefore, may be linked with how well staff members administering care are provisioned to deal with the emotional impact of shadowing. As a result, facilitating improvement in these cases may lie in finding ways to assess and increase the extent of emotional support available for staff in certain services [20].

Furthermore, some service staff seem to be more actively encouraged to carry out shadowing than others. Those asked to do shadowing exercises may not be in as much of a position to enact the required changes as others, and this may exacerbate the feeling of powerlessness amongst individuals in the organisation in terms of the ability to act on what they see [21]. Therefore, shadowing needs to take account of service structures and place a high priority on shadowing being taken on as a whole ‘team effort’ in accordance with existing team hierarchies. What makes matters worse is that the sensitivity of staff disclosure and the access limitations of the researcher make the full extent that this as an issue is difficult to assess. This reflects more broadly the dynamics of services dealing with this area and any analysis therefore should have an understanding of the sensitivity of delivering end of life care services.

Shadowing cannot be considered as a tool that is socially, politically or culturally neutral. The deployment of shadowing in services can have unforeseen additional consequences to those intended in the programme materials. It has long been held that hospital structures can play a role in the defence of staff from occupational anxieties [22]. A certain amount of social, political and cultural work had to be carried out by teams to set up, maintain, and ensure services were shadowed successfully. Many varied social, cultural and political attitudes to shadowing were found among services: some staff felt surveyed or scrutinised by being shadowed by colleagues, whilst others reacted to shadowing as if it were thrust upon them, leading us to question the extent to which shadowing could have been delegated to some staff according to how services were structured [23]. There was also an issue with some teams finding it hard to shadow because of the sensitive nature of their service (in this case community home visiting and with drug and alcohol service users). This suggests that shadowing may be difficult for some services to implement, and that shadowing may have some unforeseen social, political or cultural consequences. Therefore, the emotional impact of undertaking PFCC should be considered as part of planning and capacity building. Furthermore, the PFCC methodology should not be automatically considered to be universally applicable to all services.

## Conclusions

Many teams talked favourably of their experience of participating in the PFCC programme and gave examples of the transformative impact of the programme, particularly in terms of bringing the patient experience to the fore. What has been of concern to the research team has been the ways that the results demonstrated by shadowing in improving the quality of end of life care may be at odds with the unforeseen consequences witnessed amongst the services it was applied to during the course of the programme. The analysis has sought to disentangle issues around shadowing articulated by study participants. A key explanation given by participants for this variability was the emotional labour required to undertake the patient shadowing element of PFCC, particularly in a sensitive area of service delivery such as end of life care. Even though it is an intended consequence of shadowing to instil an emotional response to participants, there was also potential for PFCC activities to intensify the strain upon staff, particularly those at lower levels of care service hierarchies. This emphasises the importance of organisational support for staff wellbeing when carrying out service improvements in this way. The PFCC as applied to end of life care highlights the role that reconnecting with patient experience can play in service improvement for high priority areas in the NHS (NHS [24]). However, one of the reasons end of life care is a priority is due to its sensitivity, which means that improvement programmes for end of life care are potentially amongst the most difficult to carry out. As a result, while emotional responses can trigger a drive towards improvement, they can also be experienced as emotionally laborious, particularly for more junior staff within the healthcare workforce.

### Supplementary information

**Additional file 1.** Interview schedule.

**Additional file 2.** Focus group schedule.

## Data Availability

Due to the sensitivity of the data and ethical obligations study data is not generally available to people outside the evaluation team.

## Appendix

**Table 1:** Data collection methods

Methods	Data collected
Qualitative interviews with staff Phase one sites Total number of interviews 17 Number of sites 4	Visits completed to two sites (including 16 face to face interviews) One site telephone interview One site no response (data drawn from interviews with Point of Care Foundation team and documents)
Observations of events Total number of events observed 12	Observations of all learning events in phase 1 (8 observations) Observation of celebration events (2 observations) Observation of events for scale up sites (2 observations)
Qualitative interviews with four remaining sites	Telephone interviews with all sites (4 interviews)
Focus groups with sites	One focus group completed, One teleconference call
Document review	Over 700 documents in the database, documenting all stages of the program, Sorted in to the batches of: key correspondences, program set up, financing, administration, application processes, training resources, performance and program outcome data.
Patient accounts	1 completed
Interviews with coaches involved in scale up phase	5 telephone interviews
Observations of scale up phase site visits	1 completed
Interviews with staff at the scale up phase sites	4 telephone interviews, 2 informal interviews
Interviews with Point of Care Foundation PFCC team	4 completed

## Abbreviations

PFCC: Patient and Family Centred Care
PoCF: Point of Care Foundation

## Footnotes

1 The Point of Care Foundation is an independent charity that undertakes research to identify and test the most promising interventions to promote patient focused improvement in the NHS. https://www.pointofcarefoundation.org.uk/

2 The 6 steps that comprise the PFCC are: 1. Identify a care experience 2. Form a guiding council to oversee the work 3. Evaluate the current state, (e.g. shadowing, care flow mapping, patient story telling) 4. Set up working groups to take the work forward 5. Develop a shared vision for an ideal patient experience (using a driver diagram and elevator pitch) 6. Identify and take forward the individual projects. More details can be found in the Point of Care Foundation toolkit: https://www.pointofcarefoundation.org.uk/resource/patient-family-centred-care-toolkit/

## References

[1] DiGioia Anthony M., Greenhouse Pamela K., Chermak Tanya, Hayden Margaret A. (2015). A case for integrating the Patient and Family Centered Care Methodology and Practice in Lean healthcare organizations. Healthcare.

[2] DiGioia Anthony, Lorenz Holly, Greenhouse Pamela K., Bertoty David A., Rocks Suzanne D. (2010). A Patient-Centered Model to Improve Metrics Without Cost Increase. JONA: The Journal of Nursing Administration.

[3] Palliative N, End of Life Care Partnership (2015). Ambitions for Palliative and end of life care: a national framework for local action 2015–2020.

[4] Neuberger G, Aaronovitch H, Bonser P, Charlesworth-Smith J, Cox W (2013). Independent review of the Liverpool care pathway: more care.

[5] Exley Catherine (2004). Review article: the sociology of dying, death and bereavement. Sociology of Health and Illness.

[6] Willmott Hugh (2000). Death. So What? Sociology, Sequestration and Emancipation. The Sociological Review.

[7] Chochinov HM (2006). Dying, dignity, and new horizons in palliative end-of-life care. CA Cancer J Clin.

[8] Programme HFH (2013). End of life care and supporting staff: a literature review.

[9] Woo JA, Maytal G, Stern TA (2006). Clinical challenges to the delivery of end-of-life care. Prim Care Companion J Clin Psychiatry.

[10] Chung Yuh-Jin, Jung Woo-Chul, Kim Hyunjoo, Cho Seong-Sik (2017). Association of Emotional Labor and Occupational Stressors with Depressive Symptoms among Women Sales Workers at a Clothing Shopping Mall in the Republic of Korea: A Cross-Sectional Study. International Journal of Environmental Research and Public Health.

[11] Jeung Da-Yee, Kim Changsoo, Chang Sei-Jin (2018). Emotional Labor and Burnout: A Review of the Literature. Yonsei Medical Journal.

[12] Gray B, Smith P (2000). Emotional labour in the routine of nursing and in processes of death, dying and bereavement. Nurs Times.

[13] Smith P (2011). The emotional labour of nursing revisited: can nurses still care?.

[14] Dixon-Woods Mary, McNicol Sarah, Martin Graham (2012). Ten challenges in improving quality in healthcare: lessons from the Health Foundation's programme evaluations and relevant literature: Table 1. BMJ Quality & Safety.

[15] DiGioia Anthony, Greenhouse Pamela K. (2011). Patient and Family Shadowing. JONA: The Journal of Nursing Administration.

[16] Bridges Jackie, Nicholson Caroline, Maben Jill, Pope Catherine, Flatley Mary, Wilkinson Charlotte, Meyer Julienne, Tziggili Maria (2012). Capacity for care: meta-ethnography of acute care nurses' experiences of the nurse-patient relationship. Journal of Advanced Nursing.

[17] Brotheridge Céleste M., Grandey Alicia A. (2002). Emotional Labor and Burnout: Comparing Two Perspectives of “People Work”. Journal of Vocational Behavior.

[18] Brotheridge CM, Lee RT (2002). Testing a conservation of resources model of the dynamics of emotional labor. J Occup Health Psychol.

[19] Cropanzano Russell, Rupp Deborah E., Byrne Zinta S. (2003). The relationship of emotional exhaustion to work attitudes, job performance, and organizational citizenship behaviors. Journal of Applied Psychology.

[20] Brighton Lisa Jane, Selman Lucy Ellen, Bristowe Katherine, Edwards Beth, Koffman Jonathan, Evans Catherine J. (2019). Emotional labour in palliative and end-of-life care communication: A qualitative study with generalist palliative care providers. Patient Education and Counseling.

[21] Persaud Raj (2004). Faking it: the emotional labour of medicine. BMJ.

[22] Menzies IE (1960). A case-study in the functioning of social systems as a defence against anxiety: a report on a study of the nursing service of a general hospital. Hum Relat.

[23] Kelly D, Ross S, Gray B, Smith P (2000). Death, dying and emotional labour: problematic dimensions of the bone marrow transplant nursing role?. J Adv Nurs.

[24] England NHS (2014). Actions for end of life care: 2014–16.

