# Supplementary material for: The emotional labour of quality improvement work in end of life care: a qualitative study of Patient and Family Centred Care (PFCC) in England
Source: BMC Health Serv Res. 2019 Dec 2;19:923. doi: 10.1186/s12913-019-4762-1 (PMC6889450; doi:10.1186/s12913-019-4762-1)
Supplement: Supplementary file 1 — Additional file 1. Interview schedule. [file 12913_2019_4762_MOESM1_ESM.doc]

**Topic guide for Staff interviews**

1. **Introduction:**

- Go through info sheet and take consent

**Background details:**

- Gender
- Ethnic Background (self-definition)
- Job Title/Role (ask for band/grade)
- Location in department/organisational structure
- Can you tell me a bit about your background and how long you have been in your current role and employed in the organisation
- What does your role/job entail?

1. **Teams**

- In your job role, are you part of a particular team(s)?
- Can you say a little about how the team(s) you are in work in practice?
- Are there particular things that support/hinder you and your team in working effectively? (explore this point with respect to past and present issues to get sense of context).
- Are there factors that make you feel you are not part of the team? Why?

**Team context**

- How is your team working influenced by wider organisational issues?
- Who are the key actors and /influences within this context (e.g. directorate/organisational/Board level and policy contextual influences).

1. **Patient Experience/Involvement Generally:**

- Can you say something about your understanding of what it means to conduct work to improve patient experience
- Can you tell me a bit about improvement work conducted over the last 5 years (including current work), that you and your team(s) have been involved with that has had the aim of specifically improving patient experience?
- Has this work included PPI (check what they understand by this term and any differences in thinking about patient as opposed to public involvement)?
- What do you think have been the successes/ what shows you things have changed?
- What do you think patients and the public would identify as things that would help to improve patients’ and families’ experiences of care?

[Interviewer to summarise list of priorities]

1. **The process of improving care and involving patients**

- What would you consider to be the main enablers (past/present) to improve patient experiences of care? Why? How?
- What would you consider to be the main obstacles to improving patients’ experiences?
  - Explore how or whether above links specifically to PPI.
  - How might you be able to overcome barriers?
  - To what extent do you feel that you have the power to be able to improve patient experiences
  - Any trade-off in the change process
- How do you think further patient centred care can be embedded into the service?
- How do you develop your own practice to ensure that your work is patient and family centred? From successful work improving patient experience and involvement can you talk me through an example (maybe one that they feel has had most impact) of how the changes actually happened? I’m interested in what the processes and dynamics were within the teams and what happened in day to day practice? What’s the recipe for success?
- From the examples that didn’t go so well, can you again reflect on what was more difficult in the process?

1. **Measuring experiences of care and improvements**

- How do the team/ organisation collect information to understand patients’ and families’ experiences of care?
- How did these methods get developed? Who chose them and how? Are they proxies, how have they evolved (started and abandoned)? (explore if developed connected to the PFCC prog)
- Was there a particular reason for choosing these methods as opposed to other ones? (e.g. explore whether these methods were aimed at measuring particular types of changes)
- Were patients involved/ consulted in the development of these methods? If so How?
- How do you use these methods to improve patients’ experiences?
- Is the data providing you with what you need? Why/ Why not? Do these indicators show that any change is happening?

**6) Wider Context**

- Would you be able to identify any wider organisational/political issues that affect how you are able to implement improvements in patient experience including the PFCC work?
- How does the patient experience improvement work within your own service relate to wider Trust priorities and initiatives in this area? (explore relationships between different improvement initiatives at organisational level e.g. issues of responsibility/time/resources/support etc)
- How has the wider context (Trust level), affected the implementation of patient experience improvements and the development of patient centred care?
- What do you think have been the successes and what shows you things have changed (at the organisational level and team level) ?

**7) PFCC**

- Have you been involved in any way specifically with the King’s Fund work on PFCC care?

If yes, how have you been involved?

- Do you know how the PFCC programme’s work operates in practice (explore this question with respect to use of PFCC methodology (e.g. 6 steps) & practice)?
- Have patients and families been involved in this work and if so how?
- Have there been any particular issues or difficulties in carrying out the PFCC work?
- What have been the main successes in carrying out this work?
- How were the PFCC change projects decided upon?
- What measures did you use to understand whether you are succeeding in your change project goals?
  - How did you decide on these?
  - How do these measures relate specifically to patient experience measures as opposed to clinical measures?

**THANK YOU**
