# Supplementary material for: The emotional labour of quality improvement work in end of life care: a qualitative study of Patient and Family Centred Care (PFCC) in England
Source: BMC Health Serv Res. 2019 Dec 2;19:923. doi: 10.1186/s12913-019-4762-1 (PMC6889450; doi:10.1186/s12913-019-4762-1)
Supplement: Supplementary file 2 — Additional file 2. Focus group schedule. [file 12913_2019_4762_MOESM2_ESM.doc]

**Topic guide for Staff Focus Groups**

- Go through info sheet with group and take consent forms

**Before Starting gather background details of individual group members:**

Gender of group members/ Job Title/Roles of those present (ask for band/grade)/ Location in department/organisational structure of different members

1. **Introduction:**

- Go round the group in turn and ask them to introduce themselves and say a bit about their background and how long they have been in their current role and employed in the organisation, and what their role/job entails?

1. **Teams**

- Ask group if anyone would like to share any thoughts about the team(s) they are working in?
- Ask their opinion of how well their team works?
- Are there particular things that support/hinder their team in working effectively?
- Are there factors that make you feel you are not part of the team? Why?
- Is your team influenced by wider any organisational issues?
- Who are the key actors and /influences on your team? (e.g. directorate/organisational/Board level and policy contextual influences).

1. **Patient Experience/Involvement Generally:**

- Ask the group what they think is the best way improve patient experience?
- Ask group what members understand about PPI and their opinions of it(check what they understand by this term and any differences in thinking about patient as opposed to public involvement)?
- What kind of things do you think the public find important?

1. **The process of improving care and involving patients**

- Ask the group what they consider to be the main enablers (past/present) to improve patient experiences of care? Why? How?
- Ask the group the main obstacles to improving patients’ experiences?
- How do you think further patient centred care can be embedded into the service?
- Ask members to share their experiences

1. **Measuring experiences of care and improvements**

- Do the team/ organisation collect information to understand patients’ and families’ experiences of care?
- How did these methods get developed?

1. **PFCC**

- Has anyone been involved in any way specifically with the King’s Fund work on PFCC care?
- If yes, how have you been involved?
- Do you have any thoughts or opinions on the PFCC?

1. **Wrapping up**

- Does anyone have any further issues they think may be relevant?

**THANK YOU**
